# Hashimoto Encephalopathy and Thyroid Storm by Diabetic Ketoacidosis and Acute Pancreatitis: A Case Report

**DOI:** 10.7759/cureus.53659

**Published:** 2024-02-05

**Authors:** Maho Hayashi, Koji Hayashi, Machiko Miyoshi, Yasutaka Kobayashi, Mamiko Sato

**Affiliations:** 1 Internal Medicine, Fukui General Hospital, Fukui, JPN; 2 Rehabilitation Medicine, Fukui General Hospital, Fukui, JPN; 3 Health Science, Fukui Health Science University, Fukui, JPN

**Keywords:** thyroid crisis, diabetic ketoacidosis (dka), “pancreatitis”, thyroid-storm, anti-nh2-terminal of α-enolase antibody, hashimoto’s encephalopathy

## Abstract

Hashimoto encephalopathy (HE) is a rare condition related to autoimmune origin characterized by high titers of antithyroid antibodies. Steroids are effective for treatment of HE, suggesting the autoimmunity as an underlying mechanism. In addition, anti-NH2-terminal of α-enolase antibodies (anti-NAE antibodies) is useful for diagnosis of HE. This report describes a 69-year-old woman developing both HE and thyroid storm (TS), following diabetic ketoacidosis (DKA) and acute pancreatitis. She had a history of Basedow’s disease and uncontrolled type 2 diabetes mellitus, and her serum hemoglobin A1c was 10%. She complained of nausea and visited our hospital. She was diagnosed with DKA and acute pancreatitis. After admission, she went into cardiopulmonary arrest and she was diagnosed with TS after resuscitation. In addition, blood test collected during acute phase of TS revealed positive for not only anti-thyroid peroxidase (TPO) antibodies, thyroid stimulating hormone receptor antibodies and thyroid stimulating antibodies, but also anti-NAE antibodies. She was treated with intravenous steroids, potassium iodide and thiamazole under respirator and recovered sufficiently to do daily activities of life. We should keep in mind that there might be cases of HE in cases of TS presenting with central nervous system symptoms.

## Introduction

Hashimoto encephalopathy (HE) is a rare condition related to autoimmune origin characterized by high titers of antithyroid antibodies [1,2]. Steroids are effective for treatment of HE, suggesting the autoimmunity as an underlying mechanism [3]. The estimated prevalence of HE is 2.1/100,000 and HE is more common in women than in men [1,2]. Symptoms of HE are various, and may present a relapsing and remitting course and develop seizures, stroke-like episodes, cognitive decline, neuropsychiatric symptoms and myoclonus [2]. The thyroid status in HE is not consistent, but is mainly a euthyroid state or mild hypothyroidism [2]. Anti-NH2 terminal of alpha-enolase antibodies (anti-NAE antibodies) have been reported to be useful in diagnosing HE, with a specificity of 90% whereas the sensitivity is 50% [4]. In this report, we describe a rare case of both thyroid storm (TS) and HE, following diabetic ketoacidosis (DKA) and acute pancreatitis.

## Case presentation

A 69-year-old Japanese woman, who had a medical history of type 2 diabetes, developed nausea three days before admission. She had a history of smoking and alcohol (ethanol 35 ml/day). Her blood sugar control was poor, with a hemoglobin A1c (HbA1c) level of over 10% although four medications for diabetes were prescribed. On admission, her general conditions including vital signs showed mildly disturbed consciousness, BMI 20.4, blood pressure 121/80 mmHg, pulse rate 124 beats/min (regular rhythm) and body temperature 37.0°C. Physical and neurological findings revealed normal result, and lower leg edema was not pointed out. The results of blood tests revealed decreased hemoglobin and elevation of blood urea nitrogen, creatinine, potassium, glucose and HbA1c, and normal of transaminase, amylase and C-reactive protein (Table 1). Blood gas analysis disclosed metabolic acidosis under administration of one L of oxygen (Table 1). Urinary analysis revealed highly positive for urine sugar and ketone body. Abdominal plane computed tomography (CT) showed mildly enlarged head of pancreas. On the basis of elevated HbA1c and blood sugar, metabolic acidosis and highly positive for urine ketone body, she was diagnosed with DKA.

**Table 1:** Laboratory findings of blood tests and blood gas analysis on admission. Ig: immunoglobulin, pCO2: partial pressure of carbon dioxide, pO2: partial pressure of oxygen, HCO3: bicarbonate

Inspection items	Result	Reference range	Inspection items	Result	Reference range
White blood cell count	8300/μl	(3300–8600)	Creatinine	0.97 mg/dl	(0.46–0.79)
Red blood cell count	359×10⁴/μl	(386–492×10⁴)	Na	132 mmol/l	(138–145)
Hemoglobin	11.1 g/dl	(11.6–33.4)	K	6.2 mmol/l	(3.6–4.8)
Hematocrit	33.4%	(35.1–44.4)	Cl	101 mmol/l	(101–108)
Blood platelet	25.9×10⁴/μl	(15.8–34.8)	Glucose	336 mg/dl	(73–109)
Total protein	6.8 g/dl	(6.6–8.1)	Hemoglobin A1c	10.0%	(<5.5%)
Albumin	3.9 g/dl	(4.1–5.1)	C-reactive protein	0.02 mg/dl	(0.00–0.14)
Alkaline phosphatase	131 U/l	(106–322)	IgG4	14 mg/dl	(11–121)
Aspartate aminotransferase	17 U/l	(13–30)	Blood gas analysis (O2 1L, nasal)		
Alanine aminotransferase	37 U/l	(7–30)	pH	7.22	(7.38–7.46)
Lactate dehydrogenase	138 U/l	(124–222)	pCO2	20.8 Torr	(32.0–46.0)
Creatine kinase	57 U/l	(41–153)	pO2	122 Torr	(74.0–108.0)
Amylase	113 U/l	(44–132)	HCO3-	8.6 mmol/l	(21.0–29.0)
Blood urea nitrogen	39.3 mg/dl	(8.0–20.0)	Base excess	-17.4 mmol/l	(-2.0–2.0)

**Figure 1:**
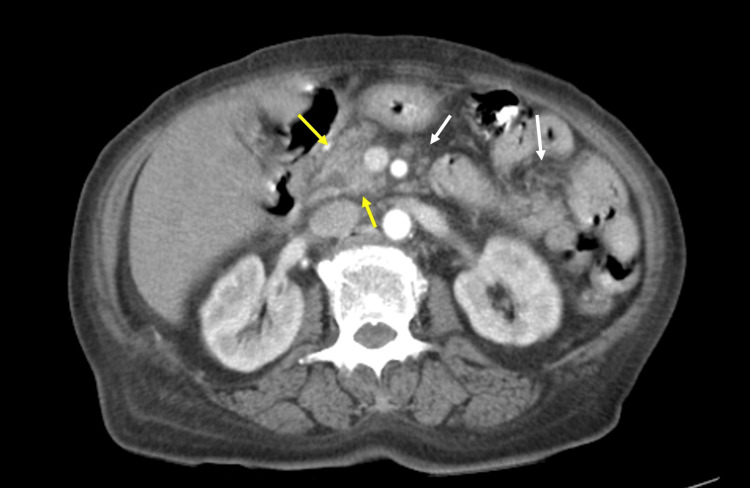
The result of contrast-enhanced abdominal computed tomography (CT). Contrast-enhanced abdominal CT showing enlargement of the pancreatic head (yellow arrows) and increased concentration of the peripancreatic fat tissues (white arrows).

She was treated with massive infusion of saline and intravenous insulin. On day two of admission, she complained of abdominal pain. Additionally, she suffered from delirium. Blood test revealed elevated amylase (950 IU/L). Abdominal contrast-enhanced CT disclosed enlargement of pancreatic head, increased concentration of peripancreatic fat tissues (Figure 1). She was diagnosed with pancreatitis and treated with gabexate mesylate. On day three of admission, thyrotoxicosis by Basedow’s disease was disclosed by blood sample on admission (decreased thyroid stimulating hormone (TSH) and elevated free T3, free T4, thyroglobulin (Tg), anti-thyroid peroxidase (TPO) antibodies, TSH receptor antibodies, thyroid stimulating antibodies) (Table 2). In addition, she developed hypoxemia and severe bradycardia suddenly followed by cardiopulmonary arrest. Fortunately, we were able to perform cardiopulmonary resuscitation successfully and she was treated with a ventilator with endotracheal intubation.

**Table 2:** The result of thyroid functions and anti-NAE antibodies on day three.

Inspection items	Result	Reference range
Free T4	≧7.77 ng/dl	(0.9–1.7)
Free T3	9.49 pg/ml	(2.3–4.0)
Thyroid stimulating hormone (TSH)	＜0.005 μIU/ml	(0.5–5.0)
Thyroglobulin (Tg)	128 ng/ml	(＜46)
Anti-thyroid peroxidase (TPO) antibodies	≧600 IU/ml	(＜16)
Anti-thyroglobulin antibodies (TgAb)	16.5 IU/ml	(＜28)
Thyroid stimulating hormone receptor antibodies (TRAb)	16.9 IU/ml	(＜1)
Thyroid stimulating antibodies (TSAb)	924%	(＜120)
Anti-NH2-terminal of α-enolase (NAE) antibodies	positive	

**Figure 2:**
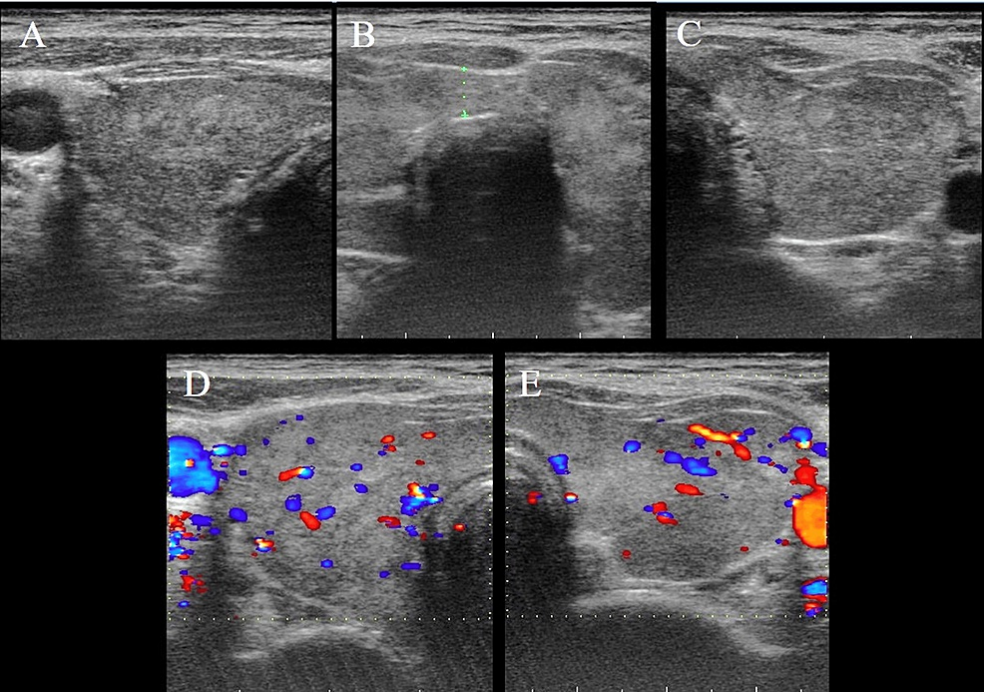
The result of thyroid ultrasonography. Thyroid ultrasonography (US) showing diffuse thyroid enlargement including the isthmus (A-C, A: right lobe, B: isthmus, C: left lobe). Color flow Doppler enhancement of the US images showing no increase in blood flow (D-E, D: right lobe, E: left lobe).

After resuscitation, she developed central nervous system (CNS) symptoms (delirium), fever (37.6 °C), tachycardia (pulse rate 120 beats/min), heart failure symptoms, and gastrointestinal symptoms in addition to thyrotoxicosis. Thyroid echocardiogram showed diffuse thyroid enlargement, with heterogeneity and internal blood flow within the normal range (Figure 2). We diagnosed definite TS based on the Japanese Thyroid Association Criteria.

We started administration of hydrocortisone (initial dose 200 mg, 100 mg every six hours thereafter), thiamazole (20 mg every six hours) and potassium iodide (50 mg every six hours) for TS. Additionally, β-blockers, calcium antagonists and diuretics were started for tachycardia-induced heart failure. After starting treatment, she had presented a fever for several days, and meropenem was administered for empirical therapy on day five. On day eight of admission, chest CT revealed pneumonia in the lower right lung field. Despite administration of meropenem, the fever did not improve, so re-study of chest CT revealed that the abnormal shadow had expanded in the lung. Assuming ventilator-associated pneumonia, we added levofloxacin and vancomycin, and multi-drug therapy of antibiotics resulted in fever resolution. Tracheostomy was performed on day 24. On day 28, she was weaned from ventilator and her consciousness was not disturbed, although sedation was discontinued. On day 47, it was revealed that serum anti-NAE antibodies were positive when TS was developed. On day 85, brain magnetic resonance imaging (MRI) revealed normal result. On the basis of these findings, she was diagnosed with HE and TS, following DKA and pancreatitis. With improvement in her clinical symptoms, steroids, thiamazole and potassium iodide were gradually tapered off. She was treated with rehabilitation therapy for six months and she was discharged from our hospital with near independence in activity of daily living, although an attention disorder was preserved due to higher brain dysfunction.

## Discussion

To the best of our knowledge, this is the first report about a case with a confirmed diagnosis of both HE and TS. Our patient developed DKA and acute pancreatitis, followed by HE and TS. The patient had uncontrolled diabetes and Basedow’s disease as a pre-existing disease. Although amylase was normal on admission, abdominal CT showed swelling of the pancreatic head, suggesting that pancreatitis had existed before admission. As for psychiatric symptoms, delirium was observed on day two. DKA is often triggered by infection, discontinuation or insufficiency of insulin therapy, cardiovascular disease, or medications including steroids, thiazides, and sodium-glucose cotransporter-2 inhibitors [5]. In addition, thyrotoxicosis may trigger DKA, and DKA may trigger TS [6]. Moreover, concurrent presentation of TS and DKA has been reported [7]. Additionally, iodinated contrast medium for CT may induce TS [8]. In our case, although the exact mechanism is unknown, the underlying thyrotoxicosis related to Basedow’s disease, uncontrolled diabetes and pancreatitis may have induced DKA, and the iodinated contrast medium and DKA may have caused TS. Interestingly, anti-NAE antibodies were positive in the serum on TS. The presence of serum anti-NAE antibodies suggested HE, because its specificity for HE is 90% [4]. However, the mechanism of HE related to TS, DKA and pancreatitis remained unclear.

HE was first reported by Lord Brain and colleagues in 1966 [9]. Since then, various cases of HE have been reported, and the clinical pictures of HE are becoming clearer. Based on numerous previous reports, the diagnostic criteria for HE has been reported [10]. Diagnostic criteria consist of six lists: 1. Encephalopathy with seizures, myoclonus, hallucinations, or stroke-like episodes; 2. Subclinical or mild overt thyroid disease (usually hypothyroidism); 3. Brain MRI normal or with non-specific abnormalities; 4. Presence of serum thyroid (thyroid peroxidase, thyroglobulin) antibodies; 5. Absence of well-characterized neuronal antibodies in serum and CSF; 6. Reasonable exclusion of alternative causes. According to diagnostic criteria, our case met at least three of six lists; thyroid disease, normal brain MRI, and positive for serum thyroid antibodies. In addition, serum anti-NAE antibodies were positive. Based on these findings, we diagnosed our patient as HE.

Diagnostic criteria for TS have been published by Japan Thyroid Association [11]. Criteria for TS consist of one ‘Prerequisite for diagnosis’ (presence of thyrotoxicosis) and five ‘Symptoms’ (CNS manifestations, fever, tachycardia, congestive heart failure, gastrointestinal/hepatic manifestations). Our case met ‘Prerequisite for diagnosis’ and four of five ‘Symptoms’, and we diagnosed our case as definite TS.

As far as we know, only one report described both HE and TS within a case [12]. In this previous report, the author mentioned a case that had a subacute progressive disorder characterized by behavioral changes, and memory and sleep disturbances, followed by an acute confusional state, fever, tremulousness, and extrapyramidal motor rigidity. Although the author thought about TS by the presence of thyrotoxicosis and fever, the author finally diagnosed HE by patient’s history and atypical clinical course for TS. Certainly, CNS symptoms are common in both HE and TS. In addition, thyroid status varies in HE [2,13], so there are some cases of HE with thyrotoxicosis like above previous report [12]. Like our case, there might be some cases of febrile HE that meet the diagnostic criteria for TS. In such cases, measurement of anti-NAE antibodies might be useful for diagnosis of HE.

There are three major limitations in our report. First, we could not evaluate the responsiveness to steroids or changes in consciousness level. These are useful for confidence in the diagnosis of HE [13]. However, we treated our patient with respirator and sedatives, which made them unclear. Second, we could not obtain physiological or radiographic tests during the hyperacute stage including electroencephalogram, brain MRI and brain perfusion scintigraphy. Initially, we assumed that the patient had TS, but it was proved that the patient was positive for anti-NAE antibodies after the acute phase. Therefore, we could not plan physiological or radiographic tests. Third, there is no previous report about the measurement of anti-NAE antibodies in TS. CNS symptoms are developed by not only HE but also TS itself. In addition, although thyroid status of typical HE cases is euthyroid or mild hypothyroidism, HE with thyrotoxicosis has been reported like our case. Therefore, it should be noted that some cases of TS are difficult to distinguish from HE. Whereas we diagnosed HE based on a positive anti-NAE antibody, further studies are needed about anti-NAE antibodies in TS cases.

## Conclusions

To the best of our knowledge, this is a first report about a case with a confirmed diagnosis of both HE and TS. In some cases of TS with CNS symptoms, it might be difficult to distinguish from HE. Additionally, thyroid function in HE is variable and non-constant, and some cases of HE are accompanied by thyrotoxicosis. In particular, febrile HE with thyrotoxicosis may meet the diagnostic criteria for TS. In such cases, measurement of anti-NAE antibodies might be useful for diagnosis of HE. It is required to accumulate more cases to reveal the underlying mechanism regarding complications of HE and TS. 
